# A catalytic hairpin amplification platform triggered by near-infrared light and logic assembly for sensitive detection of microRNAs

**DOI:** 10.1039/d5na00775e

**Published:** 2025-11-11

**Authors:** Ruiqi Chen, Bin Qiu, Chen Chen, Mingyuan Chen

**Affiliations:** a The Department of General Medicine/Geriatrics, Fujian Medical University Union Hospital Fuzhou 350001 China; b College of Chemistry, Fuzhou University Fuzhou 350108 China; c Department of Clinical Nutrition, the First Affiliated Hospital, Fujian Medical University Fuzhou 350000 China ciece3@qq.com; d Department of Clinical Nutrition, National Regional Medical Center, Binhai Campus of the First Affiliated Hospital, Fujian Medical University Fuzhou 350000 China; e Department of Hepatobiliary Surgery, Fujian Institute of Hepatobiliary Surgery, Fujian Medical University Union Hospital Fuzhou 350001 China chenmingyuan99@163.com

## Abstract

Research has shown that miRNA-21 levels increase during vascular aging, making it a promising biomarker for vascular aging. Therefore, developing sensitive miRNA-21 detection techniques is crucial for early diagnosis, therapeutic intervention, and prognosis assessment of vascular aging. This work developed an enzyme-free fluorescence sensing platform based on upconversion nanoparticles (UCNPs) and a catalytic hairpin assembly (CHA), which uses 808 nm light-controlled switches and fluorescence resonance energy transfer (FRET) signal regulation for highly sensitive and specific quantification of miRNA-21. First, the loaded DNA hairpins and the UCNPs were released under weakly acidic conditions due to the disintegration of the organic framework material (ZIF-8). Under 808 nm irradiation, when the analyte miRNA-21 is present, the DNA hairpin (H1) undergoes specific unwinding, triggering CHA, leading to signal recovery of the fluorescent group on another DNA hairpin (H2) and quantitative detection of miRNA-21. The degree of fluorescence restoration of the sensor exhibited a good linear correlation with the miRNA-21 concentration in the range of 0.5–25 nM, and the limit of detection reached 0.128 nM. Moreover, this method exhibits excellent analytical performance in the detection of serum samples, with spiked recovery rates ranging from 98.2% to 102.8%. These data demonstrate that the light-controlled sensing platform has significant merits, such as high detection sensitivity, strong specificity, and minimal background interference, providing a reliable method for the rapid quantification of miRNA-21 in complex matrices.

## Abstract 2

An NIR-active CHA biosensor for the quantification of miRNA-21.

## Graphical abstract

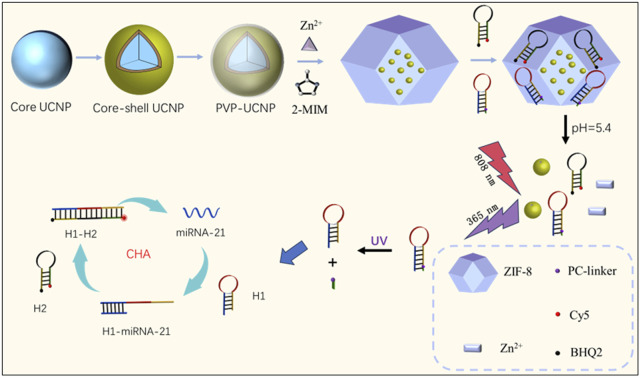

## 1 Introduction

Vascular aging refers to the degenerative alterations in structure and function that occur as blood vessels gradually lose their original organic energy with age, and is a special type of organ aging.^1^ Vascular aging is an important pathological basis for coronary atherosclerotic heart disease, hypertension, cerebrovascular disease and many other diseases.^2^ MicroRNAs (miRNAs) are small noncoding RNAs, about 18–25 nucleotides long, that regulate target gene expression and are involved in critical cellular processes like proliferation, differentiation, and apoptosis.^3–5^ Several studies have demonstrated that the dysregulation of miRNA expression is tightly associated with the incidence and progression of vascular aging and related diseases, making it a highly promising diagnostic biomarker for vascular aging.^6^ For example, miRNA-21 is abnormally expressed in vascular aging and has been recognized as a biomarker for the diagnosis and prognosis of vascular aging.^7^ Therefore, the development of highly sensitive and specific miRNA detection methods is highly important for the diagnosis and subsequent treatment of vascular aging in clinical practice.

Currently, the approaches for quantifying miRNAs include Northern blotting,^8,9^ microarray,^10,11^ and real-time quantitative polymerase chain reaction (RT-qPCR).^12,13^ Although these strategies have certain sensitivity and precision in miRNA detection, they have several limitations, such as expensive instruments, complex operations, and high reagent costs.^14^ With biosensing technology advancing quickly in recent years, numerous new miRNA detection techniques have been developed, such as electrochemiluminescence biosensors,^15,16^ photoelectrochemical biosensors,^17,18^ and fluorescence biosensors.^19,20^ Among these, fluorescent biosensors have gained significant attention due to their advantages, including high repeatability, rapid response, and simple instrumentation.^21^ Currently, most fluorescent biosensors employ ultraviolet-visible (UV-vis) light for the excitation source.^22^ However, UV-vis light has high energy and can cause light damage to biological samples.^23,24^ In addition, for UV-vis excitation windows, biomolecules produce strong spontaneous fluorescence and light scattering, which restricts their use in biosensing applications.^25^ Hence, developing new fluorescent biosensors is essential for accurate and sensitive detection of miRNAs in biological samples.

Upconversion nanoparticles (UCNPs), a unique class of luminescent materials, can convert low-energy near-infrared (NIR) light into higher-energy UV-vis emissions, exhibiting anti-Stokes fluorescence properties.^26^ Since UCNPs are excited by NIR light (typically at 808 nm or 980 nm), they offer dual advantages: minimizing photodamage associated with UV-visible excitation while simultaneously reducing autofluorescence interference.^27^ Therefore, constructing UCNP-based fluorescent biosensors for miRNA detection is a reliable strategy. Moreover, owing to the low abundance and high sequence similarity of miRNAs, these methods generally combine signal amplification strategies, such as catalytic hairpin assembly (CHA),^28,29^ hybrid strand reaction (HCR),^30,31^ and pivot-mediated strand switching amplification reactions, to improve detection sensitivity.^32,33^ Among them, CHA is widely used in UCNP-based biosensing because of its high catalytic efficiency, low cost, and ease of operation.^34,35^ Although these methods have achieved great progress in miRNA analysis, most of the sensing probes are “always active”, resulting in false-positive signals and a lack of detection accuracy.^36^ Therefore, combining an NIR-activated CHA signal amplification strategy to construct fluorescent biosensors is expected to achieve sensitive and accurate detection of miRNAs. However, relatively little research has been conducted on this strategy in the field of biosensing.

**Scheme 1:**
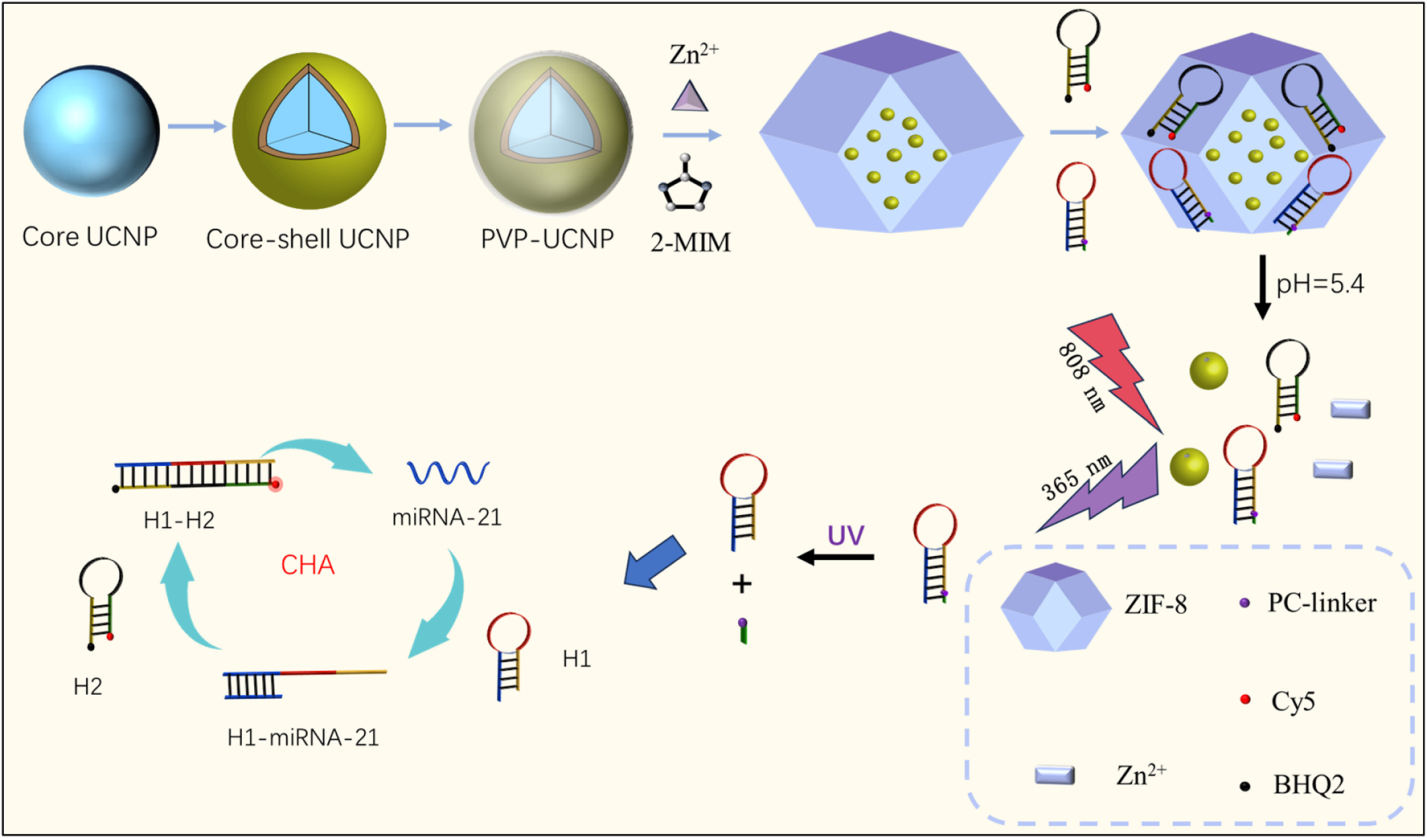
Schematic design of an NIR-active CHA biosensor for the quantification of miRNA-21.

Here, a CHA signal amplification sensing platform based on NIR light activation was constructed by embedding photolytic bonds into target miRNA recognition elements, transforming the sensor from a “passive trigger” to a “controllable activation” state for sensitive and specific quantification of miRNA-21 (as shown in Scheme 1). First, the loaded DNA hairpins and the UCNPs were released under weakly acidic conditions due to the disintegration of the organic framework material (ZIF-8). When irradiated at 808 nm, UCNPs can transform NIR light into UV-vis light, disrupt PC-linkers on the DNA hairpin (H1), and selectively expose the target binding site, effectively avoiding nonspecific triggering in complex biological media. H1 is recognized by the target miRNA and initiates CHA amplification. Simultaneously, another DNA hairpin (H2)-labelled BHQ-2 and Cy5 at its two termini is turned on, causing Cy5 to move away from BHQ-2, resulting in the fluorescence changing from “OFF” to “ON” and releasing the target miRNA to catalyze the amplification of the next assembly signal. The light-responsive switch mechanism biosensor constructed in this project not only significantly reduces background interference but also achieves high-sensitivity detection of miRNAs in complex biological samples through CHA signal amplification.

## 2 Experimental

### 2.1 Instruments and agents

The morphological and structural characteristics of the nanomaterials were analyzed by using a JEM-2100F transmission electron microscope (TEM). X-ray powder diffraction (XRD) patterns were obtained *via* Cu K_α_ radiation (*λ* = 0.15418 nm) on a Miniflex 600 diffractometer in Japan. Fourier transform infrared spectroscopy (FTIR) analysis was conducted using a Nicolet 5700 infrared spectrometer. Zeta potential measurements were performed with a Nano-ZS90 zeta potential analyzer. The ion content of the solution was quantified by inductively coupled plasma optical emission spectrometry (ICAP 7400, Thermo Scientific). The valence states of the elements were determined *via* X-ray photoelectron spectroscopy (XPS, ESCALABQXI). The upconversion excitation and emission spectra were recorded using a FluoroMax-4 spectrofluorometer with an external 808 nm CW laser (2 W) excitation source.

YbCl_3_·6H_2_O, GdCl_3_·6H_2_O, TmCl_3_·6H_2_O, NdCl_3_·6H_2_O, oleic acid (OA), 1-octadecene (ODE), phosphate-buffered salt solution (PBS, pH 7.4), and 4-hydroxyethyl piperazine ethanesulfonic acid (HEPES) were purchased from Aladdin (Shanghai, China) Co., Ltd NaOH, HCl, anhydrous ethanol, NH_4_F, methanol, 2-methylimidazole (2-MIM), polyvinylpyrrolidone (PVP), cyclohexane, dimethylformamide (DMF), and Zn(NO_3_)_2_·6H_2_O were purchased from China Hai Aladdin Company. All oligonucleotide sequences and PC-linker (photocleavable) were provided by Shanghai Sangon Biotechnology Co., Ltd in China. The DNA and RNA chains are as follows:

H1: 5′-TAGCTT/iPCLink/ATCAGACTGATGTTGATATATTTTTTTTTTTTTCAACATCAGTCTGATAAGCTA3′

H2: 5′-Cy5-TATATTTTTTTTTTTTATCAGACTGATGTTGAAAAAAAAAAAAATATATCAACATC-BHQ-2-3′

miRNA-21: 5′-UAGCUUAUCAGACUGAUGUUGA-3′

miRNA-155: 5′-UUAAUGCUAAUCGUGAUAGGGGU-3′

miRNA-222: 5′-AGCUACAUCUGGCUACUGGGU-3′

### 2.2 Synthesis and modification of the core–shell UCNPs

First, a mixture of ODE (15 mL) and OA (6 mL) containing GdCl_3_·6H_2_O (0.30 mmol), TmCl_3_·6H_2_O (0.01 mmol), and YbCl_3_·6H_2_O (0.69 mmol) was heated to 160 °C under vacuum. After cooling to 40 °C, a methanol solution with 148 mg of NH_4_F and 100 mg of NaOH was introduced, followed by heating to 150 °C and stirring for 30 min. Afterward, the temperature was increased to 300 °C and held for 1.5 h. The entire process was carried out in a helium environment. Following cooling to room temperature, 20 mL of ethanol was introduced, and the mixture was centrifuged at 13 000 rpm for 10 min. The core UCNPs (NaGdF_4_:Yb,Tm) were then purified *via* three ethanol washes before being resuspended in 10 mL cyclohexane.

A mixture of ODE (15 mL) and OA (6 mL) (containing 0.48 mmol of GdCl_3_·6H_2_O, 0.24 mmol of NdCl_3_·6H_2_O and 0.08 mmol of YbCl_3_·6H_2_O) mmol was heated to 150 °C under vacuum conditions and stirred for 0.5 h. Upon cooling to 40 °C, the as-synthesized NaGdF_4_:Yb,Tm was introduced, and the temperature was raised to 110 °C to remove cyclohexane by evaporation. Upon re-cooling to 40 °C, a methanolic solution of NH_4_F (148 mg) and NaOH (100 mg) was added dropwise, followed by 30 min of stirring. Subsequently, the temperature was raised to 110 °C in nitrogen for 15 min, and then the temperature was elevated to 300 °C in nitrogen for 1 h. After cooling to room temperature, 20 mL of ethanol was added, the mixture was centrifuged at 13 000 rpm for 10 min, washed with ethanol three times, and redispersed in 10 mL of cyclohexane. Therefore, NaGdF_4_:Yb,Tm@NaGdF_4_:Yb,Nd (the core–shell UCNP) with OA ligands was obtained.

The abovementioned UCNPs (0.04 mmol) were suspended in 5 mL of cyclohexane. 5 mL of chloroform solution containing 0.01 M was added. After shaking, a flocculent precipitate formed. The mixture underwent centrifugation at 10 000 rpm for 10 min, the supernatant was removed, the precipitate was dissolved in 2 mL of DMF, 5 mL of cyclohexane was added, and the mixture was centrifuged under identical conditions (10 000 rpm, 10 min) to isolate the precipitate. The precipitate was dispersed in 5 mL of ethanol. Under vigorous agitation, a chloroform solution (15 mL) containing PVP (0.02 mmol) was added dropwise, followed by continuous stirring for 24 h. Post-reaction centrifugation yielded the precipitate. Methanol was used to remove more than PVP, and thus, the aqueous phase PVP-UCNP was obtained.

### 2.3 Synthesis of the UCNP@ZIF-8 nanomaterials

The as-prepared PVP-UCNPs were initially dispersed in 4 mL of methanol and stirred at room temperature for 30 min. Subsequently, 5 mL of a methanolic solution of Zn(NO_3_)_2_·6H_2_O was introduced dropwise to the above mixed solution. After dropwise addition, the mixture turned milky white. The reaction mixture was continuously stirred at ambient temperature for 24 h. When crystal growth was complete, the mixture was centrifuged. The resulting white precipitate was dispersed in methanol, centrifuged, and washed multiple times with methanol to remove the excess organic ligands. The white powder obtained was the UCNP@ZIF-8 nanocomplex.

### 2.4 Construction of the sensor based on UCNP@ZIF-8@DNA

DNA sequences (H1 and H2) were heated to 95 °C for 10 min followed by gradual cooling to room temperature, facilitating hairpin formation. 4 µL of H1 (10 µM) and 4 µL of H2 (10 µM) were mixed with 40 µL of the above-prepared UCNP@PVP@ZIF-8 in 64 µL of HEPES buffer (125 mM HEPES, 685 mM NaCl, pH 7.4) and incubated at room temperature for 1 h. The unbound DNA strands were eliminated to obtain UCNP@ZIF-8@DNA.

### 2.5 Fluorescence detection of miRNA-21

First, 160 µL of PBS buffer (pH 5.4) was incorporated into 40 µL of the above-prepared UCNP@ZIF-8@DNA, after which diverse concentration gradients of miRNA-21 were added (final concentrations of 1, 5, 7.5, 10, and 25 nM). The reaction mixture was incubated at 37 °C for 40 min, followed by 20-min NIR irradiation (808 nm) to initiate the amplification cascade. The resulting solution was incubated at 37 °C for 90 min. Fluorescence emission spectra (660–700 nm) were acquired under 643 nm excitation, with triplicate measurements performed for each experimental group.

### 2.6 Analysis of actual samples

Prior to miRNA-21 detection using the UCNP@ZIF-8@DNA system, serum samples were 20-fold diluted with PBS (pH 7.4). miRNA-21 quantification was performed *via* the standard addition method, following identical detection protocols as described previously. Recovery studies were subsequently conducted through spiking experiments with known concentrations of miRNA-21.

## 3 Results and discussion

### 3.1 Characterization of the synthesized nanomaterials

**Fig. 1:**
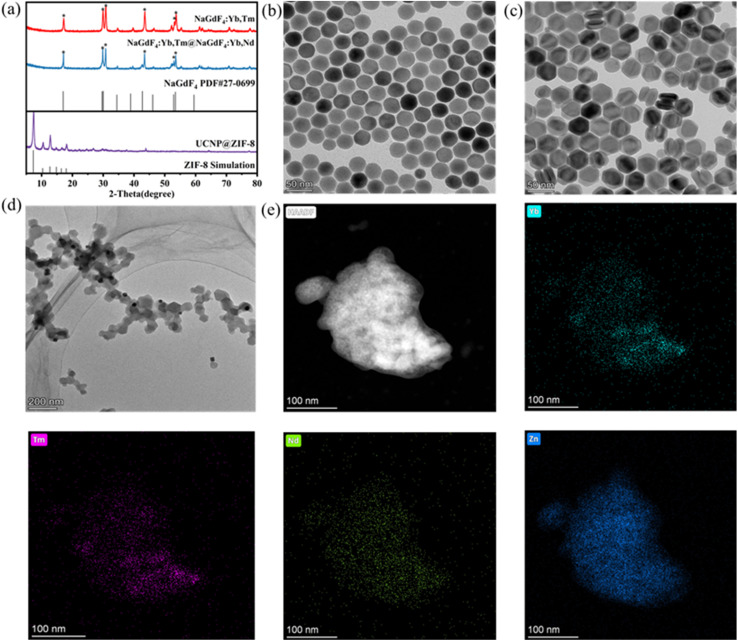
(a) XRD pattern of the nanomaterials; (b) TEM image of NaGdF_4_:Yb,Tm; (c) TEM image of NaGdF_4_:Yb,Tm@NaGdF_4_:Yb,Nd; (d) TEM image of UCNP@ZIF-8; (e) elemental distribution characterization.

First, XRD, TEM, and EDS were used to characterize the morphology and structure of the fabricated nanomaterials. As shown presented in Fig. 1a, the diffraction peaks (2*θ*) of NaGdF_4_:Yb,Tm and NaGdF_4_:Yb,Tm@NaGdF_4_:Yb,Nd coincided with the classical peaks of the NaGdF_4_ hexagonal phase at 17.005°, 29.655°, 30.002°, 42.717°, 52.88°, and 53.446°, and no other impurities were detected. These results indicate that UCNPs with high crystallinity were prepared. After coating with ZIF-8, the classical NaGdF_4_ diffraction peak disappears. Moreover, the diffraction peak (2*θ*) of the ZIF-8 crystal coincided with the diffraction peak positions of the standard card of ZIF-8 at 7.30°, 10.35°, 12.70°, 14.80°, 16.40°, and 18.00°, indicating that the UCNPs were encapsulated inside ZIF-8. Fig. 1b shows TEM images of the core UCNPs, which are highly monodisperse, with an average size of 25.29 nm. After further coating the NaGdF_4_ shell, the resulting core–shell UCNPs were dumbbell shaped, and their average size increased to 40.55 nm (Fig. 1c). The TEM images of UCNP@ZIF-8 (Fig. 1d) show that the prepared material is a regular dodecahedron and is coated with dumbbell-shaped core–shell UCNPs. Finally, EDS analysis (Fig. 1e) was used to determine the presence of elements, such as Yb, Tm, Nd, and Zn. The above results indicate that the core UCNPs, the core–shell UCNPs and UCNP@ZIF-8 were prepared.

**Fig. 2:**
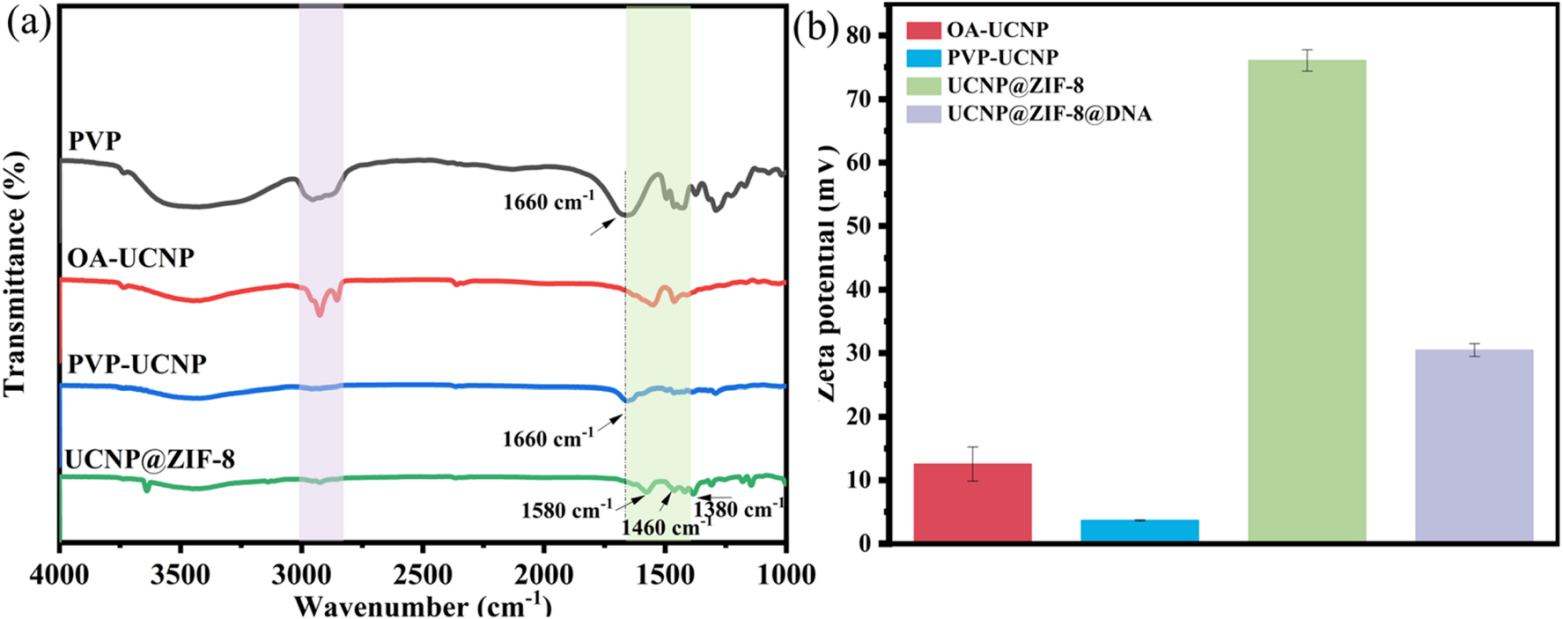
(a) FTIR spectra of the nanomaterials; (b) zeta potentials of the nanomaterials.

Second, FTIR and potential methods were employed to analyze the molecular structure, functional moieties, and surface charge of the prepared nanomaterial. Fig. 2a shows that after surface modification of the UCNPs with PVP, the disappearance of –CH_2_ stretching vibrations (3000–2800 cm^−1^) attributed to OA molecules, coupled with attenuated –COO^−^ vibrational intensity (1600–1400 cm^−1^) and emergence of a carbonyl stretch at 1660 cm^−1^, conclusively demonstrates successful PVP functionalization. Following ZIF-8 encapsulation of UCNPs, FTIR spectroscopy revealed the emergence of a characteristic absorption band at 1580 cm^−1^, corresponding to the C=N stretching vibration of imidazole ligands in the zeolitic framework. In addition, in-plane antisymmetric bending vibrations and symmetric bending vibrations of the C–H bond on the imidazole ring of 2-methylimidazole were observed at 1460 cm^−1^ and 1380 cm^−1^, respectively. The above results demonstrate the successful encapsulation of ZIF-8. Fig. 2b shows that the potential potentials of the OA-UCNP and PVP UCNPs are 12.6 and 3.6 mV, respectively. After PVP modification, the surface potential of the nanomaterial significantly decreased, further revealing the removal of OA surface ligands and successful grafting of PVP onto the UCNP surfaces. After coating with ZIF-8, the surface potential of the nanomaterial increased to 76.1 mV, which could originate from surface-exposed Zn^2+^ ions in the ZIF-8 framework, confirming the coating of ZIF-8 on the UCNPs. Following DNA hairpin conjugation, the zeta potential decreased significantly to 30.5 mV, attributable to the negatively charged phosphate backbone of DNA. This substantial reduction in surface potential verifies successful nucleic acid immobilization on the nanomaterial.

**Fig. 3:**
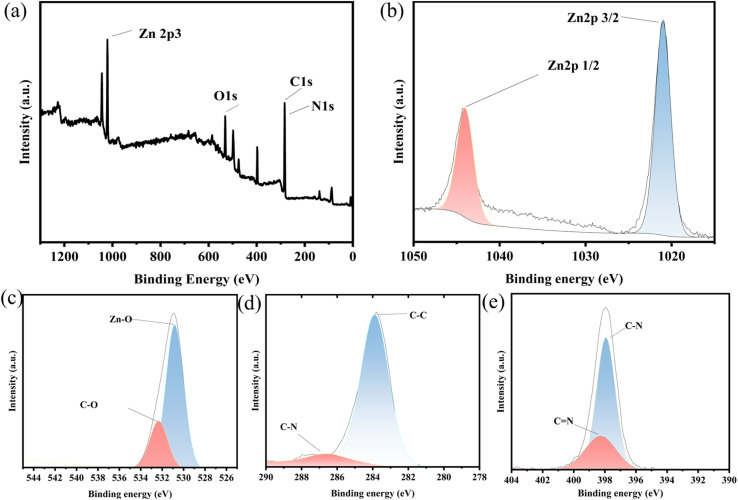
XPS spectra of UCNP@ZIF-8: (a) total spectrum; (b) Zn 2p peak; (c) O 1s peak; (d) C1s peak; (e) N 1s peak.

Finally, XPS was employed to characterize the elemental composition and chemical states in UCNP@ZIF-8. The survey spectrum (Fig. 3a) confirms the existence of C, N, O, and Zn. The binding energies of Zn 2p are located at 1021 eV and 1044.3 eV (Fig. 3b), which are assigned to Zn 2p_1/2_ and Zn 2p_3/2_, respectively. The O 1s region is shown in Fig. 3c, with the peak at 531 eV associated with typical metal–oxygen coordination and the peak at 532 eV attributed to C–O single bonds. The XPS spectrum of C 1s can be divided into two peaks: the binding energy at 283.9 eV can be attributed to the characteristic peak of C–C, and the characteristic peak of C–N at 286.5 eV corresponds to the carbon hydrogen bond in the imidazole ring of the ligand (Fig. 3d). The binding energies of N 1s are all approximately 398 eV, which is attributed to the characteristic peak of pyridine-type nitrogen in the imidazole ring (Fig. 3e). Multiple characterization techniques including XRD, TEM, FTIR, and potential analysis measurements, indicate that the expected UCNP@ZIF-8 was successfully prepared.

### 3.2 Principle and feasibility of NIR-activated CHA

**Fig. 4:**
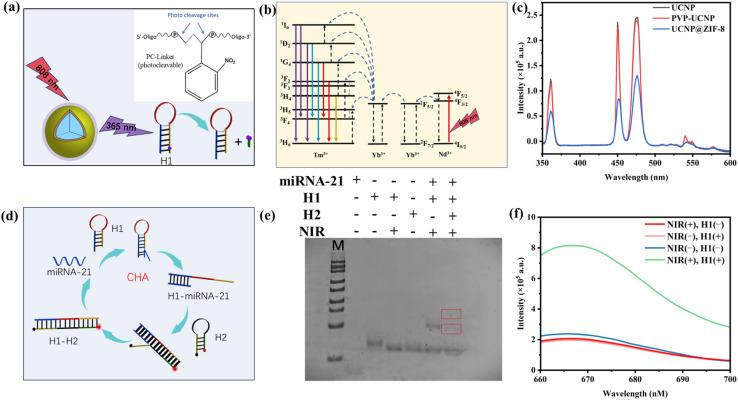
(a) Schematic diagram of NIR light control and PC-linker (photocleavable) chemical structure; (b) energy transfer trajectory of the UCNPs; (c) fluorescence spectra of the nanomaterials; (d) schematic diagram of CHA; (e) PAGE images of the different detection systems; (f) fluorescence spectra of miRNA-21 by NIR and H1 in different combinations in the presence of target substances and H2.

In this work, a CHA signal amplification sensing probe based on NIR light activation was constructed for sensitive and specific detection of miRNA-21. When irradiated at 808 nm, UCNPs can transform NIR light into UV-vis light, disrupt PC-linkers on the DNA hairpin (H1), and selectively expose the target binding site. The PC-linker (photocleavable) chemical structure is shown in Fig. 4a. H1 is recognized by the target miRNA and initiates CHA amplification (Fig. 4d). Therefore, we synthesized UCNPs doped with Yb^3+^, Tm^3+^, and Nd^3+^, and their energy transfer trajectory (Nd^3+^ → Yb^3+^ → Tm^3+^) is shown in Fig. 4b. The fluorescence spectrum (Fig. 4c) revealed that the obtained UCNPs emitted 365 nm ultraviolet light under 808 nm excitation, which provided the necessary conditions for the destruction of the PC-linker on the DNA hairpin (H1) and the activation of CHA. However, after coating with ZIF-8, the luminescence intensity decreased, further verifying that UCNP@ZIF-8 was successfully prepared.

The CHA amplification process can be understood as a form of logical assembly in which hairpins H1 and H2 interact according to a defined sequence of conditional rules. Initially, in the absence of the target, H1 and H2 retain their hairpin conformations, preventing signal generation. Upon introduction of the target, H1 opens, exposing a sequence domain that enables H2 to bind and thereby restores the fluorescence signal. Crucially, H2 subsequently releases the target, allowing the cycle to repeat and achieve signal amplification. From a logical assembly perspective, each step represents a conditional operation: the opening of H1 depends on the presence of the target, the hybridization of H2 relies on the activated H1, and the continuation of the cycle requires the successful release of the target. In this manner, the CHA reaction exemplifies a conditionally and sequentially organized assembly, demonstrating how molecular components can be programmed to perform logic-guided amplification (Fig. 4d). To verify the results of CHA signal amplification, PAGE was conducted, and lanes 1–6 included miRNA-21, H1, H1 + NIR, H2, H1 + miRNA-21 + NIR and H1 + H2 + miRNA-21 + NIR, respectively. As shown in Fig. 4e, in lanes 2 and 3, the UCNPs emit 365 nm ultraviolet light and disconnect the PC-linker of H1 after irradiation at 808 nm. Lanes 1, 3, 4, and 5 show that miRNA-21 fully hybridizes with H1, which disconnects the PC-linker. From lanes 1, 3, 4, and 6, it can be inferred that the binding site of H2 is exposed when H1 binds to miRNA-21, resulting in the complementary base pairing between H1 and H2 to form a more stable double-stranded structure. To further validate the realizability of the proposed photoactivatable and CHA amplification design, the fluorescence recovery of Cy5 at 664 nm was observed with and without H1 and NIR irradiation. As displayed in Fig. 4f, CHA be can triggered only when both NIR and H1 are used as inputs, resulting in the recovery of red fluorescence. Without the participation of H1, CHA cannot be triggered, so the fluorescence of Cy5 is almost undetectable. Furthermore, when H2 is absent, the sole binding of H1 to the target is insufficient to enable H2 to interact with H1 and subsequently restore fluorescence, and H2 cannot trigger the cycling process. However, a comparison of the fluorescence emission intensities of Cy5 with and without NIR light irradiation revealed that the UCNPs did not emit UV light without irradiation at 808 nm. Therefore, CHA cannot be triggered, and Cy5 fluorescence is almost undetectable. The above results further verify the feasibility of CHA design activated by NIR light.

### 3.3 Acid response of UCNP@ZIF-8

**Fig. 5:**
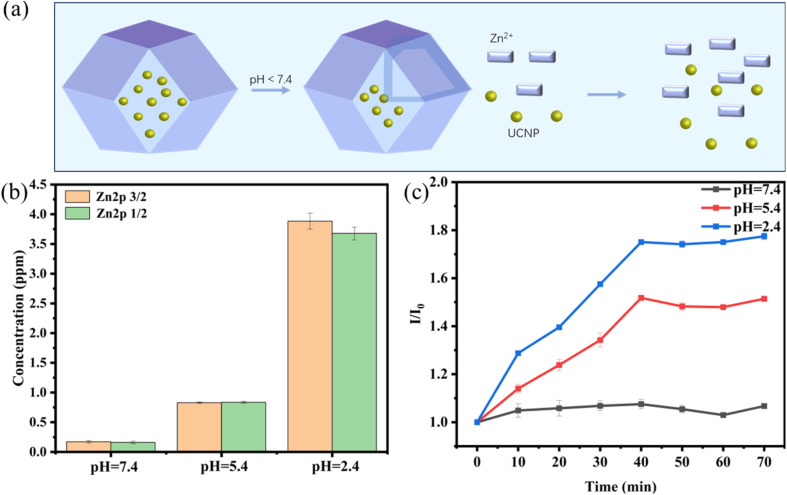
(a) Schematic diagram of UCNP@ZIF-8 cracking under acidic conditions; (b) Zn^2+^ concentration in different pH solutions; (c) recovery of the luminescence intensity of the UCNPs under different pH environments.

Fig. 5a shows a schematic diagram of UCNP@ZIF-8 cracking under acidic conditions. When encountering an acidic environment, ZIF-8 cracks, releasing the coated UCNPs, H1, and H2. To verify this phenomenon, the Zn^2+^ concentration in the solution was quantified *via* an inductively coupled plasma emission spectrometer after UCNP@ZIF-8 cleavage at varying pH levels. As illustrated in Fig. 5b, when the solution changed from neutral (pH 7.4) to acidic (pH 2.4), the Zn^2+^ concentration in the solution rose from 0.171 ppm to 3.884 ppm, verifying the pH-responsive behavior of ZIF-8. In addition, with the cleavage of the ZIF, the coated UCNPs are gradually released, so the pH-responsive properties of the ZIF can also be verified by monitoring UCNP luminescence. As illustrated in Fig. 5c, the temporal recovery profiles of UCNP luminescence intensity exhibit significant pH dependence. The emission intensity attained maximum values within 40 min and subsequently maintained plateau levels for an additional 30 min period. In addition, the variation in the luminescence recovery signal was highly dependent on the pH, and as the pH decreased, the luminescence recovery efficiency gradually increased. The above results indicate that ZIF-8 has acid cleavage properties and can therefore be used to release adsorbed UCNPs and DNA hairpin (H1 and H2) acidic environments.

### 3.4 Analytical parameter optimization

**Fig. 6:**
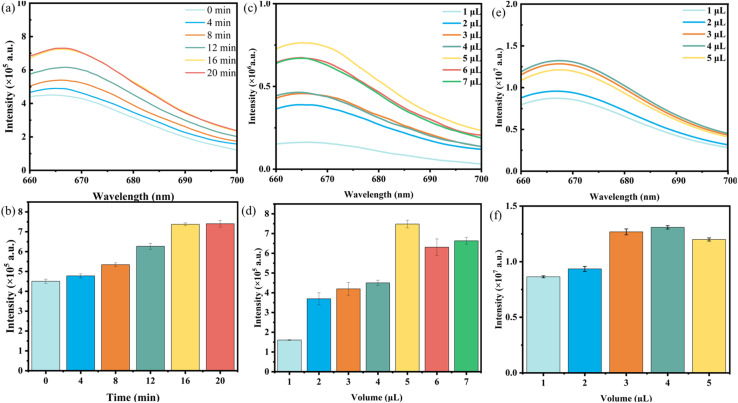
(a and b) Effects of different durations, (c and d) H1 amounts, and (e and f) H2 amounts on the fluorescence intensity at 664 nm.

Prior to quantification of the target, several experimental parameters affecting the progress of CHA and the recovery of Cy5 fluorescence, such as the illumination time at 808 nm, the amount of H1 and the amount of H2, were optimized. First, the effects of different durations (0–20 min) of 808 nm laser irradiation on the fluorescence of Cy5 at 664 nm were investigated. Fig. 6(a and b) shows that with increasing illumination time, the fluorescence intensity of Cy5 gradually increased, reaching its highest value at approximately 20 min and tending to stabilize. Therefore, 20 min is chosen as the optimal lighting time. The effects of the H1 amount (Fig. 6(c and d)) and H2 amount (Fig. 6(e and f)) on the fluorescence signals were also studied, and 5 µL (1 µM) and 4 µL (1 µM) were selected as the optimal amounts for H1 and H2, respectively, for subsequent experiments.

### 3.5 Sensing platform performance evaluation

**Fig. 7:**
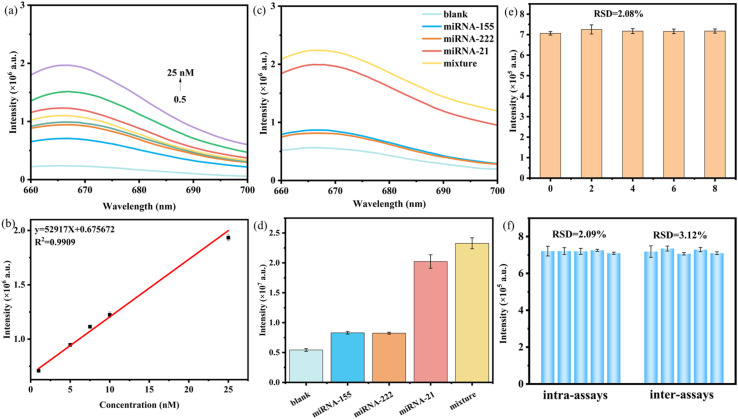
(a) Fluorescence response curves of probes to miRNA-21 at varying concentrations; (b) linear relationship between fluorescence intensity and miRNA-21 concentration; (c and d) specificity of the proposed approach for detecting miRNA-21; (e) stability test within 8 days; (f) reproducibility of the proposed biosensor.

To examine the ability of a catalytic hairpin amplification platform triggered by near-infrared light and logic assembly for detecting target miRNA-21, the constructed fluorescent probe was added to target miRNA-21 with varying concentrations (final concentrations of 1, 5, 7.5, 10, and 25 nM) under optimized experimental conditions. As presented in Fig. 7a, as the concentration of the target miRNA-21 increased, the fluorescence intensity of the sensing system gradually increased at 664 nm. Fig. 7b demonstrates a quantitative relationship between fluorescence response and miRNA-21 concentration, exhibiting excellent linearity (*R*^2^ = 0.9909) across 0.5–25 nM. The regression equation *y* = 529 17*x* + 0.675672 (*y*: fluorescence intensity; *x*: miRNA-21 concentration) mathematically describes this dose-dependent response. The limit of detection (LOD) was determined to be approximately 0.128 nM (S/N = 3). Compared with several reported methods (Table 1), although the LOD of the proposed method is slightly lower or comparable, it does not require enzyme involvement and has a shorter detection time, indicating that the designed nanoprobe is suitable for simple, rapid, and sensitive miRNA detection.

**Table 1:** Comparison of miRNA detection performance

Material	Time (min)	Amplification strategy	Linear range (nM)	Detection limit (nM)	Reference
DNA	75	APE1/CHA	2.5–40	0.22	37
DNA	80	CHA	0.5–20	0.441	38
UCNP	120	CHA	0.5–4.5	0.44	39
DNA/AgNCs	180	CHA	0.2–20	0.2	40
AuNP-FAM	120	DSN	0.3–8	0.3	41
UCNP/DNA/PC-linker	60	NIR/CHA	0.5–25	0.128	This work

Next, to evaluate the specificity of the proposed detection method for the target miRNA-21, we examined the influence of competing miRNAs on the detection system's fluorescence response. The results in Fig. 7(c and d) clearly show that the probe designed in this work hardly produces a signal response to interfering miRNAs (such as miRNA-155 and miRNA-222) that coexist with miRNA-21. Only in the presence of the analyte miRNA-21 did the sensing platform have significant signal recovery, indicating that the probe designed in this work has good recognition specificity for the analyte miRNA-21 and has greater potential for application in complex biological environments.

In addition, the stability of the fabricated biosensor was evaluated by detecting miRNA-21 every 48 h over an 8-day period, with three replicates analyzed at each interval. As illustrated in Fig. 7e, during the testing period, there was no significant fluctuation in the fluorescence response of the biosensing platform to 5 nM miRNA-21 at 664 nm, and the relative standard deviation (RSD) of the change in the fluorescence signal was 2.08%. To further assess reproducibility, both intra-assay and inter-assay variations were investigated and Fig. 7f shows the RSD values of 2.09% and 3.12% for fluorescence signal changes, respectively. These results indicate that the biosensor exhibits excellent stability and repeatability for miRNA-21 detection.

### 3.6 Actual sample analysis

We evaluated the applicability and reliability of a catalytic hairpin amplification platform triggered by near-infrared light and logic assembly by detecting miRNA-21 in serum samples *via* UCNP@ZIF-8@DNA biosensors. Under the optimized experimental conditions mentioned above, spiked recovery experiments were conducted by adding different concentrations of miRNA-21 (1, 3, and 5 nM). Table 2 shows that the spiked recovery rate in the actual samples was between 98.2% and 102.8%, with a relative standard deviation (RSD, *n* = 3) of less than 4.6%. The UCNP@ZIF-8@DNA biosensor demonstrates robust miRNA-21 quantification capability in serum samples, exhibiting exceptional environmental adaptability in complex biological matrices while maintaining high detection accuracy.

**Table 2:** Determination of miRNA-21 in serum samples

Sample	Added (nM)	Found (nM)	Recover (%)	RSD (%)
Serum1	1	0.982 ± 0.037	98.2	3.7
	3	3.066 ± 0.046	102.2	1.5
	5	4.982 ± 0.068	99.6	1.3
Serum2	1	0.986 ± 0.046	98.6	4.6
	3	3.084 ± 0.068	102.8	2.2
	5	4.955 ± 0.047	99.1	0.9

## 4 Conclusions

In summary, a nonenzyme-mediated CHA signal amplification platform based on near-infrared light triggering and logic assembly was established for the sensitive quantification of miRNA-21. In the sensing system, ZIF-8 is used to load DNA deliver H1 and H2 for cyclic amplification of CHA. Compared with traditional detection methods, this sensor can effectively avoid false positive signals and enhance the precision of detection results. In addition, UCNPs doped with Yb^3+^, Tm^3+^, and Nd^3+^ were utilized to convert near-infrared light into ultraviolet light. The NIR light-controlled switch was turned on to trigger the CHA amplification reaction while avoiding spontaneous fluorescence and light scattering from biomolecules *via* UV excitation, which can reduce environmental interference. However, there are still some limitations in the current research. Therefore, in future work, we will further construct a multi-miRNA detection platform to achieve accurate early diagnosis of vascular aging by replacing miRNA complementary sequences in sensing probes (*i.e.*, replacing miRNA-21 complementary sequences in existing probes with specific complementary sequences of other miRNAs related to vascular aging).

## Ethical statement

Serum samples were collected from Fujian Medical University Union Hospital (Fuzhou, China) with written informed consent obtained from all participants. All animal procedures were performed in accordance with the Guidelines for Care and Use of Laboratory Animals of Fujian Medical University Union Hospital and approved by the Animal Ethics Committee of Fujian Medical University Union Hospital.

## Author contributions

Ruiqi Chen, Chen Chen and Mingyuan Chen designed research. Ruiqi Chen, Bin Qiu and Chen Chen performed the experiments. Ruiqi Chen, Bin Qiu and Mingyuan Chen analyzed data. All author wrote and revised the manuscript.

## Conflicts of interest

The authors declare that they have no known competing financial interests or personal relationships that could have appeared to influence the work reported in this paper.

## Data Availability

The authors confirm that the data supporting the findings of this study are available within the article.
